# Breast ironing: A brief overview of an underreported harmful practice

**DOI:** 10.7189/jogh.11.03055

**Published:** 2021-03-27

**Authors:** Fikrejesus Amahazion

**Affiliations:** College of Arts and Social Sciences, Adi Keih, Eritrea

Harmful traditional practices are particular forms of violence against women and girls which are defended on the basis of tradition, culture, religion, or superstition by some community members. Around the world, millions of girls and women are impacted by a range of harmful traditional practices, including child and/or forced marriage, bride kidnapping or abduction, female genital cutting/mutilation (FGC/M), dowry or bride prices, female infanticide, and son preference, among others [1].

While many of these practices have generated great interest and received considerable coverage in recent years, there has been comparatively little research conducted on or attention paid to breast ironing, a harmful practice mainly performed on girls and women in parts of Africa south of the Sahara. This paper provides a brief overview of the practice, reviews the factors driving the practice, discusses its implications for health and rights, and also presents a series of recommendations.

## BREAST IRONING

Breast ironing, sometimes referred to as breast flattening, is a harmful practice that generally involves the repetitive pounding, pressing, ironing, rubbing, or massaging of a pubescent girl’s breasts, often using hard or heated objects, in order to attempt to stop or delay them from growing or developing, make them flatter, or make them disappear. The practice can include the use of a variety of objects, such as heated grinding stones, cast-iron pans, ladles, hammers, wooden pestles or spatulas, spoons, brooms, or electric irons. Other objects that may be utilized include pits of black fruits, coconut shells, plantain peels, and certain leaves or plants (which are believed to possess medicinal or healing qualities). Breast ironing may also involve tightly wrapping or tying bandages, elastic compresses, cloths, or belts around young girls’ chests [2,3].

As with a number of other harmful traditional practices (eg, FGC/M), breast ironing is typically performed by female familial relatives (eg, mother, sister, aunt, grandmother, nanny, or another female guardian). Generally, the practice is maintained as a secret between girls and their mothers or other guardians. At times, traditional midwives, healers, and shamans may perform the practice, which can provide them with a regular income (or other general reimbursements) and elevated social status.

To date, data and empirical studies on breast ironing have been extremely scarce, thus limiting broad understanding about its extent or general prevalence. However, in a 2006 press release, the United Nations Population Fund listed breast ironing as one of “five under-reported stories relating to gender-based violence” [4]. Countries where the practice is believed to occur include Benin, Burkina Faso, Cameroon, the Central African Republic, Chad, Côte d’Ivoire, Guinea-Bissau, Guinea-Conakry, Kenya, Nigeria, Togo, South Africa, and Zimbabwe. Notably, a 2005 nationwide study conducted in Cameroon estimated that approximately 25% of girls and women had undergone the procedure [2]. Moreover, there is no evidence indicating that breast ironing is correlated with religion, ethnicity, wealth, or formal education [3].

Due to migration, it is likely that breast ironing may also be practiced in other African countries or among African diaspora populations in the West (eg, North America and Western Europe). In recent years there have been a number of news reports of the practice occurring within immigrant communities in the United Kingdom [5,6].

## IMPORTANT FACTORS BEHIND BREAST IRONING

A considerable body of research has illustrated that there is no one single factor to account for various harmful traditional practices [7-10]. Similarly, breast ironing is driven by a number of different factors.

One important factor underlying the practice is that it is regarded as a form of “protection” [11-13]. Specifically, it is performed as a way to help disguise the onset of puberty in girls, which it is believed will help to deter male attention and protect them from sexual harassment, assault, exploitation, and rape or sexually transmitted diseases. Notably, a number of demographic surveys and empirical studies conducted in countries where breast ironing is believed to take place have shown that women and girls are often at risk of sexual harassment, sexual violence, and sexually transmitted diseases [14-18].

Another contributing factor for the practice is child marriage, which continues to be highly prevalent in many parts of Africa. A large body of research has documented the adverse economic, social, demographic, psychological, and reproductive health consequences of child marriage for child brides, their families, and their communities. For example, child marriage severely curtails educational and employment opportunities for girls, and it can have a long-term, adverse impact on their quality of life [19-21]. With the development or growth of breasts in pubescent girls often socially signaling or denoting that they are “ready” for marriage in some communities, breast ironing is seen as a way to help ensure that they are prevented from undergoing child marriage.

Breast ironing is also motivated by longstanding traditional norms, entrenched sociocultural attitudes, and widespread gender inequality. Specifically, in many societies, deeply ingrained patriarchal norms contribute to rigid gender roles and privilege or ascribe higher status to men and lower status to women. Cultural ideals of femininity promote modesty, while female sexuality is often regarded as shameful and something that must be repressed, hidden, and denied. Moreover, chastity and virginity at marriage are considered as highly important elements in a girl’s personal and family honor. Breast growth and development in girls is regarded as intricately tied to their transition into womanhood and signifies the emergence of their sexuality. Accordingly, breast ironing aims to uphold cultural ideals about gender roles, social relations, and appropriate sexual behaviors. It is an attempt to retain control over women’s bodies and sexuality, and seen as a way to ensure chastity and prevent early pregnancy, premarital sexual relations, or children out of wedlock – all of which can significantly tarnish or dishonor a family’s social status or standing.

**Figure:**
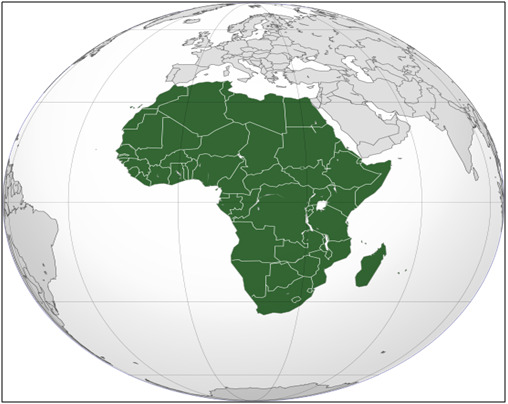
Photo: Breast ironing has been found in some parts of Africa and African diaspora populations (Image source: Wikimedia)

## HEALTH AND RIGHTS ISSUES

While data and findings from long-term health-related or medical studies are scarce, breast ironing is believed to expose girls and women to a number of significant risks. For example, breast ironing can violate a young girl’s physical integrity and put victims at risk for severe pain or discomfort, swelling or tissue damage, irritation, burns, scars, bruising and discoloration, deformities, wrinkles, and spots on their breasts or chests [2,6,22,23]. Additionally, the practice may contribute to emotional and psychological issues (eg, fear, breast dissymmetry leading to low self-esteem, shame, or anxiety about one’s body, or loss of trust and confidence in relatives or caregivers).

As with a number of other harmful traditional practices, breast ironing may be considered as a violation of basic and fundamental human or gender-related rights, as set out in multiple universal and regional human rights instruments including: the *Universal Declaration of Human Rights*; the *United Nations Convention on the Elimination of all Forms of Discrimination Against Women*; the *United Nations Convention on the Rights of the Child*; the *African Charter on Human and Peoples’ Rights*; the *Protocol to the African Charter on Human and Peoples’ Rights on the rights of Women*, and the *African Charter on the Rights and Welfare of the Child*.

Generally, these instruments affirm the fundamental rights and freedoms of every human being and they commit governments to protect women and change discriminatory, harmful, or exploitative practices and laws. However, breast ironing represents a denial of the dignity and integrity of the individual, and denies girls and women the right to live free from gender discrimination or violence. Furthermore, it is often carried out without the consent of the female involved, and it restricts victims’ agency, decision-making, and control over their own bodies, lives, and sexuality.

## MOVING FORWARD

Moving forward, greater attention toward breast ironing is critically required in order to increase awareness and better protect the rights and interests of girls and women. There are a number of possible steps that can be taken in this direction. For example, more and methodologically rigorous population-based studies and data collection are necessary to estimate the prevalence, burden, and impact of breast ironing and provide critically relevant information for governments, policymakers, health care professionals, international organizations, and communities.

As well, those working with young girls and women, such as health care workers and school officials, should be trained to identify the signs and symptoms of girls who may be at risk of or have already undergone breast ironing. Resources and services should also be established to provide appropriate support, treatment, rehabilitation, and counseling to girls and women who have undergone breast ironing. As well, midwives, traditional healers, and others who may rely on performing breast ironing for economic support or general reimbursements should receive help to find alternative income generating activities.

Importantly, governments can create supportive legislative and regulatory frameworks. For example, breast ironing can be added to existing laws outlining prohibitions of harmful traditional practices (eg, FGC/M and child marriage), or states can enact stand-alone comprehensive, progressive legislation on breast ironing that is inline with international legal and human rights norms, principles, and standards. Furthermore, the application and enforcement of existing laws against child marriage and sexual violence must be improved, particularly since they represent key motivating factors for breast ironing.

It is also vital to increase public education and awareness about breast ironing, as well as promote better understanding of prevention of pregnancy and HIV or other sexually transmitted infections. For example, community outreach programs and public workshops can raise awareness about breast ironing and its negative impact on girls’ and women’s physical, emotional, and psychological health. In order to facilitate local buy-in and promote broad dissemination, awareness campaigns should involve respected religious figures and local leaders, such as village chiefs, tribal elders, and political leaders, and can also utilize local media outlets and general communication opportunities. Information about breast ironing can also be integrated into life skills and comprehensive sexuality education curricula taught to young people.

Finally, gender equality must be promoted and the deeply entrenched patriarchal norms, values, beliefs, and attitudes underlying breast ironing must be firmly challenged. Societies where breast ironing is practiced must critically examine and reform the systematic societal structures that institutionalize male physical, social, and economic power over women, oppressing them and restricting them to subjugated positions in society. Women and girls’ empowerment should be encouraged, while their inherent value and basic rights must be respected and protected. Communities need to transform the cultural paradigms and social frameworks where females turn to the harmful practice of breast ironing as a protective coping mechanism for sexual violence or child marriage. Importantly, men and boys should also be engaged in order to change their attitudes, beliefs, and behaviors related to power, control, discrimination, and violence.
